# Implications of the cattle trade network in Cameroon for regional disease prevention and control

**DOI:** 10.1038/srep43932

**Published:** 2017-03-07

**Authors:** Paolo Motta, Thibaud Porphyre, Ian Handel, Saidou M. Hamman, Victor Ngu Ngwa, Vincent Tanya, Kenton Morgan, Rob Christley, Barend M. deC. Bronsvoort

**Affiliations:** 1 The Roslin Institute, University of Edinburgh, Easter Bush Campus, EH25 9RG, United Kingdom; 2 Royal (Dick) School of Veterinary Science, University of Edinburgh, Easter Bush Campus, EH25 9RG, United Kingdom; 3 Institute of Agricultural Research for Development, Regional Centre of Wakwa, Ngaoundere, B.P. 454, Cameroon; 4 School of Veterinary Medicine and Sciences, University of Ngaoundere, Ngaoundere, B.P. 454, Cameroon; 5 Cameroon Academy of Sciences, Yaound´e, B.P. 1457, Cameroon; 6 Institute of Ageing and Chronic Diseases, University of Liverpool, Leahurst Campus, CH64 7TE, United Kingdom; 7 Institute of Infection and Global Health, University of Liverpool, Leahurst Campus, CH64 7TE, United Kingdom; 8 NIHR Health Protection Research Unit in Emerging and Zoonotic Infections, University of Liverpool, Liverpool, L69 3BX, United Kingdom

## Abstract

Movement of live animals is a major risk factor for the spread of livestock diseases and zoonotic infections. Understanding contact patterns is key to informing cost-effective surveillance and control strategies. In West and Central Africa some of the most rapid urbanization globally is expected to increase the demand for animal-source foods and the need for safer and more efficient animal production. Livestock trading points represent a strategic contact node in the dissemination of multiple pathogens. From October 2014 to May 2015 official transaction records were collected and a questionnaire-based survey was carried out in cattle markets throughout Western and Central-Northern Cameroon. The data were used to analyse the cattle trade network including a total of 127 livestock markets within Cameroon and five neighboring countries. This study explores for the first time the influence of animal trade on infectious disease spread in the region. The investigations showed that national borders do not present a barrier against pathogen dissemination and that non-neighbouring countries are epidemiologically connected, highlighting the importance of a regional approach to disease surveillance, prevention and control. Furthermore, these findings provide evidence for the benefit of strategic risk-based approaches for disease monitoring, surveillance and control, as well as for communication and training purposes through targeting key regions, highly connected livestock markets and central trading links.

Movements within and between populations are a central driver of disease dynamics defining the patterns of interactions and the susceptibility to the spread of a wide range of infectious agents^1^,^2^,^3^,^4^. Livestock trade is of particular importance, since pathogens can be transmitted over long distances via movement of infectious animals. Understanding the structure of livestock contacts and studying the routes, volumes, frequency and the risks associated with animal movement represents a prerequisite for effective animal and zoonotic disease surveillance and control. Most industrialised countries have implemented animal identification, registration and tracing systems to enhance strategic and targeted approaches for disease surveillance, to develop early warning systems for outbreak detection and for more informed control measures^5^. However, in most lower income countries, where the presence of endemic diseases represents an obstacle for the development of animal trade and the improvement of the livestock sector as a whole, there is still limited information on livestock movements with no systematic recording systems^6^.

Currently some of the world’s most rapid urbanization is taking place in West and Central Africa, where the population is poised to quadruple in size by the end of this century^7^. As *per capita* consumption of animal-source foods is projected to continue rising and even to accelerate in the short-medium term^8^, the volume of livestock trade is likely to expand in this region. In West and Central Africa livestock have traditionally been raised in semi-arid regions to be traded in forested zones and urban areas^9^. These long distance, and often cross-border, movements are still central to the livelihoods of many pastoral communities, traders and intermediaries^10^. Despite variations in the level of organization across countries, livestock trade often occurs via local, regional or national livestock auctions and commodity markets. These markets represent major contact points in livestock populations which not only facilitate trade and social interactions but also play critical roles in the dispersal of infectious diseases in diverse farming systems^1^,^2^,^11^,^12^,^13^,^14^,^15^.

In Cameroon, the livestock sector is rapidly evolving toward a more cattle-oriented, market-orientated system and represents an important source of revenue for about 30% of the rural population^16^. While organized around traditional smallholders, the Cameroonian livestock industry depends on a well established and structured livestock market system to facilitate trade. Therefore, the flow of animals within the country can be described as a network which considers markets as nodes that are linked by the transfer or movement of animals. In general, the structural characteristics of such networks are known to influence the potential dissemination of infectious agents^17^,^18^,^19^. For example, networks in which most connections (or links) in the network are held by a small number of nodes are known to ease the spread of infectious diseases^17^,^20^. Of particular interest are the so-called “small-world” networks. Small-world networks are characterized by the tendency of neighboring nodes to cluster together and, simultaneously, by the presence of few long-distance connections linking local clusters standing far apart within the network. This high clustering can facilitate the rapid local spread of socially transmitted diseases^19^, whereas the long ties can result in the dissemination of pathogens to distant areas of the network^11^,^21^. The number and size of subgroups of nodes, or “components”, which compose these networks would therefore affect the cohesion of the overall structure and, consequently, the potential impact of infections over the network. Knowing the size and location of these components is not only critical to assess the potential of the network to generate large epidemics^22^,^23^,^24^, but also provides a basis to identify nodes which are key for the cohesion of the network. In an epidemiological context, this information is critical^25^,^26^,^27^, particularly by facilitating the design of targeted communication, monitoring, surveillance and control programmes and increasing the cost-effectiveness of interventions.

In this paper we present the first formal study of the livestock trade network in Cameroon using a social network analysis approach. Combining the collection of official trading data and a questionnaire based survey at livestock trading points throughout Western and Central-Northern Cameroon carried out between October 2014 and May 2015, we present the analysis of the annual cattle movements through livestock markets at a national level. The objectives of this paper were to (1) characterize the current formal cattle movements through the network formed by the livestock trading system, (2) to quantify the flow of cattle within this network and (3) to identify key markets for practical surveillance, control and communication interventions.

## Methods

### Study site

**Figure 1:**
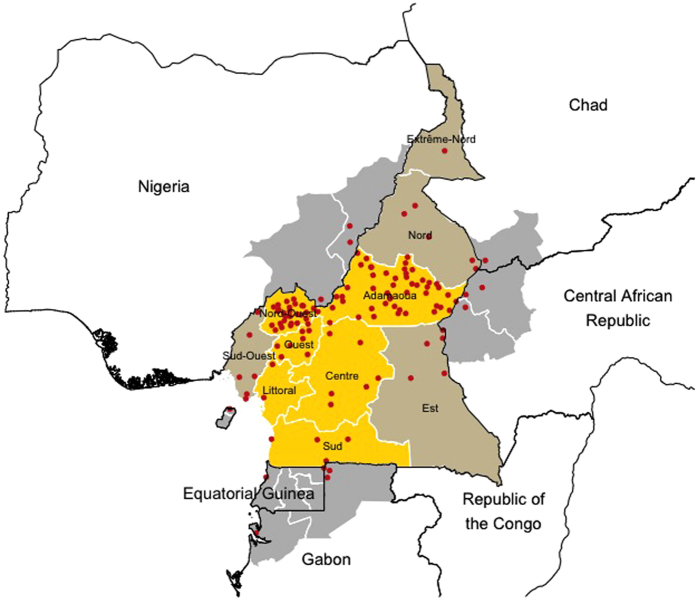
Study area and locations of the livestock markets included in the analysis. The Regions in yellow highlight the areas where data collection was carried out. Regions in gold are the areas of Cameroon that were not visited during the study but for which were identified trading links with livestock markets localized in the sampling areas. In grey are highlighted Regions of neighboring countries where livestock markets outside Cameroon are connected with the trade network (Generated using R statistical software^40^ (version 3.2.3) using the *raster, rgdal* and *ggplot2* packages, and shp files obtained from the GADM database of Global Administrative Areas (source: www.gadm.org)^49^.

The study area comprised the West, North-West and Adamawa Regions and represents the main cattle production areas in Cameroon. This study focused mainly on these three administrative Regions but major markets of the Littoral, Central and South Regions were also included (Fig. 1).

The Adamawa Region is mainly a pastoral highland of approximately 64,000 *km*^2^ considered to be the main livestock production area of Cameroon with an official cattle population of about 1,250,000^28^ and known to be both a source and a destination of transhumant herds originating from other areas of the country during the dry season (October - April). The North-West Region is a mountainous area hosting about 450,000 cattle, while the West Region has a flatter landscape with a smaller cattle population of about 160,000 animals^28^.

### Cattle markets system in Cameroon

Cattle marketing in Cameroon involves transactions in conventional infrastructures owned by the Government where herders, traders, butchers and other stakeholders carry out central activities for the commercialization of animals. These cattle markets mostly occurred once a week, with few large markets open 6 days a week. Traders, are important actors and tend to acquire cattle in sub-regional or regional markets and commercialize them along the trading chain towards the national or border markets. Once acquired by traders, cattle are moved and traded along this chain of markets until they are sold and exit the trading system.

### Data collection

#### Cattle markets identification

For each of the study Regions, a list of the livestock markets was obtained from the relevant Regional Delegation of the Ministry of Livestock, Fisheries, and Animal Industries (MINEPIA) and it was evaluated in consultation with regional livestock experts. Information relative to the commercial connections during the previous 12 months was gathered from all markets included in the MINEPIA registers. However, preliminary data collection from these markets and interviews with local veterinary officials indicated that the list was incomplete. All markets that were not listed in the MINEPIA registers but were identified through their commercial connections and were located within the three study Regions (Adamawa, West and North-West), were added to our data collection framework and subsequently visited (see Supplementary Section A). Markets identified by local veterinarians which were not listed in the MINEPIA registers and which were located outside the three administrative Regions of core interest in this study were kept in the network but not visited (with the exception of the three major markets in Yaounde, Douala and Kye-ossi). Within the three administrative Regions of core interest (Adamawa, West and North-West Regions) a total of 52 markets were listed in the MINEPIA register. The preliminary analysis of the information relative to the commercial connections of the listed markets during the previous 12 months identified an additional 7 markets within the three Regions (total of 59 markets). Furthermore, 68 markets were identified outside these three study Regions: 46 were listed by MINEPIA in Cameroon (including the 3 national markets located in Yaounde and Douala, the two major urban centres of Cameroon, and in Kye-ossi, a border town in the South Region neighbouring with Gabon and Equatorial Guinea); 10 were identified by local veterinarians to be within Cameroon and an additional 13 markets in the 5 neighbouring countries. Overall, the data comprised of 127 markets located in Cameroon and five neighbouring countries (Fig. 1). Details of the number of markets and their identification process are reported in the Supplementary Material Section A.

#### Market records collection and questionnaire-based survey

From October 2014 to May 2015 official market records were collected in all livestock markets listed in the MINEPIA market register (*n* = 52) as well as those not present in the register but located within the study area (*n* = 7). Together with official market transaction records reports were also collected. Data for a 12-month period from September 2013 to August 2014 was extracted from this official documentation. In Cameroon, transactions are recorded on paper and handwritten. Market records were therefore scanned using a portable wireless scanner and a smart phone, manually transcribed to an electronic databases by two persons separately, and cross-checked for discrepancies. When erroneous or missing records were identified, original scans were re-examined and data re-entered in the database.

Although the number of animals traded during each market day was consistently reported, the origins and destinations were not recorded consistently between Regions. To confirm the data obtained from these sources and gather information where missing, semi-structured interviews were conducted with the veterinary officials and market managers using French and English (Cameroonian official languages) and two local languages (Fufulde and Cameroonian Pidgin). Interviewees were asked to identify all the origins and destinations of traded animals, as well as the weights of these connections, defined as the number of traded animals per month. Data sources and data quality evaluation are shown in the Supplementary Material Section B.

To evaluate changes in the characteristics and number of animal traded throughout the year, records were classified as occurring during either the dry or the rainy season over the study period. However, as the definition of these seasons varied between respondents, participants were asked to define the months corresponding to these periods.

### Markets Networks Construction

Data regarding numbers, origins and destinations of cattle movements between markets were extracted from the database combining the information from the data sources (market records, official reports and the survey) and then used to build a weighted static directed network. Markets formed the nodes of the network and the links between markets were defined as the movement of at least one animal from an origin market to a destination market. These contacts were described according to the direction of the movement and weighted by the number of cattle that were traded over this link.

Data were aggregated over a 12-month period and stratified by the two seasonal intervals, October to April for the dry season and May to September for the rainy season. All network measures and summary statistics were estimated on the annual and seasonal networks to assess seasonal variations in both network centrality and connectivity metrics.

### Network Analysis

Description of the cattle trade network in Cameroon was carried in two steps: (1) we looked at the topology of the network, particularly by assessing if the cattle trade network has a small-world structure; and (2) by identifying key markets solely based on their node centrality measures. All network and node centrality measures used in this analysis are defined in Table 1.

#### Network topology

Topology of the observed cattle trade network in Cameroon was first described by using various network-level metrics, including density, reciprocity, degree assortativity, diameter, average path length (PL) and clustering coefficient (CC). A network is said to have a small-work structure if its CC is significantly higher than that computed from a random network of equivalent size and connections (that is with same number of nodes and links), while showing a lower value of PL^29^. We therefore assessed if the observed empirical cattle trade network has a small-world structure by comparing its CC and PL measures with those computed from a set of 1000 randomly-generated networks.

Node-level metrics were further computed to evaluate the centrality of each individual market within the network. These measures included in- and out-degree, betweenness and eigenvector centrality (Table 1). These centrality metrics were computed over the weighted directed network, accounting for the weights of the connections^30^. The distribution of node degree was assessed following the guidelines described by Clauset *et al*.^31^ (See Supplementary Section E).

#### Nodes characteristics and Key-Actors Analysis

Identification of key markets was investigated through a correlation analysis between nodes centrality measures. Markets were classified depending on their relationship between eigenvector centrality and betweenness centrality. This analytic approach was derived from the study of the relationships among network metrics by Valente *et al*.^32^ and was previously applied to identify central actors of covert social networks by Conway^33^. The residuals of the linear regression were used to identify central markets as the ones displaying a greater predicted value than the observed one. The size of the regression residuals, in combination with the results from the correlation analysis, were used to identify key markets and define their functional roles within the network.

#### Cohesive analysis

Here, we explored the overall network connectivity and structural features of the network by carrying out successive cohesive sub-group analyses. First, a core-periphery analysis^34^ was carried out to identify densely-connected core nodes and sparsely-connected periphery nodes. We then assessed the distribution of components present in the network and identified the largest connected components. In this analysis, “components” represent subgroups of nodes that are maximally connected between each other. However, components may be of two types: accounting (or not) for the direction of links in the network components may be either “strongly” and “weakly” connected. Strong components are subgroups of nodes in which a node can be reached from every other considering the directionality of links, whereas weak components are subgroups of nodes for which directionality of the links is disregarded. The size of the largest, also named “giant”, strongly (GSCC) and weakly (GWCC) connected components were then computed. It is worth noting that GSCC and GWCC are considered as proxy measures for lower and upper bounds of maximal epidemic size, respectively, for epidemics spreading in the considered network^22^,^23^,^24^.

Finally, we explore the distribution of communities present in the network. While components analysis uncovers nodes that are reachable following a contact path, communities detection identifies sub-groups of nodes densely connected to each other but sparsely connected with nodes in other communities^35^. The number and composition of node communities were characterised using the Rosvall and Bergstrom, or Infomap, community detection algorithm^36^,^37^,^38^. This method, also called “maps of random walks”, is a flow-based clustering approach using a random walk as a proxy for the flow on a network to identify the shortest of the random walks on the network to reveal its community structure. Among various methods assessed the Rosvall and Bergstrom method provided the closest community structure in relation to the actual geographical localization of these markets and to the presence of natural barriers favoring the creation of more densely connected sub-groups of markets (see Supplementary Section C).

### Network vulnerability and resilience

A percolation analysis was implemented to assess the vulnerability of the network to the targeted removal of nodes. Briefly, this analysis consisted of measuring the impact of progressively removing nodes, one after another, in the decreasing order of a given node centrality measure, on the structure of the network. In this analysis, we considered that the removal of nodes consisted of implementing trade restrictions, vaccination or further animal testing, which would prevent disease to be transmitted to other nodes and spread^39^. In this context, the resilience and the vulnerability of the cattle trade system in Cameroon to the targeted manipulation of the structure was assessed upon the cohesiveness of the network, thereby mimicking the impact of focused interventions on the network. Here, the cohesiveness of the network was measured in several ways, by computing at each removal step the sizes of the GSCC, of the GWCC and of the biggest community in the cattle trade network, as well as the total number of communities present in the remaining networks. Node centrality measures used in the analysis to drive the removal processes were in-degree, out-degree, betweenness and eigenvector and of the results from the key-actor analysis.

To better assess the impact of targeted interventions over the network connectivity the structural vulnerability of the network to strategic removal of central nodes was compared with the random removal of nodes. The targeted removal driven by the different centrality measures used in this study, including the results from the key-actor analysis, was compared with the median and the 95% range of 1000 simulations of random removals. Additionally, interventions targeting the most critical markets in the network functionality, as identified by the key-actors analysis, were compared with approaches targeting markets belonging to the top 10*th* percentiles of each centrality measure. All analysis and graphics were performed in R statistical software^40^ (version 3.2.3) using the *igraph, tnet, fitdistrplus, glm, raster, rgdal, circlize* and *ggplot2* packages.

### Ethical Statement

This research has been funded by the University of Edinburgh through a Principal’s Career Development Scholarship and was authorised by the Ministry of Livestock, Fisheries and Animal Industries (MINEPIA), and approved by the Cameroon Academy of Sciences. In the United Kingdom approval was given by the Veterinary Ethical Review Committee of the Royal (Dick) Veterinary School (University of Edinburgh).

All methods were performed in accordance with the relevant guidelines and regulations and informed consent was obtained from all subjects. Interviewers were trained to provide the information regarding the consent process to be communicated to the participants. Prior to interviewing, the study objectives, procedures and the content of the questionnaires were also explained to the participants.

## Results

### Summary statistics and general network properties

**Figure 2:**
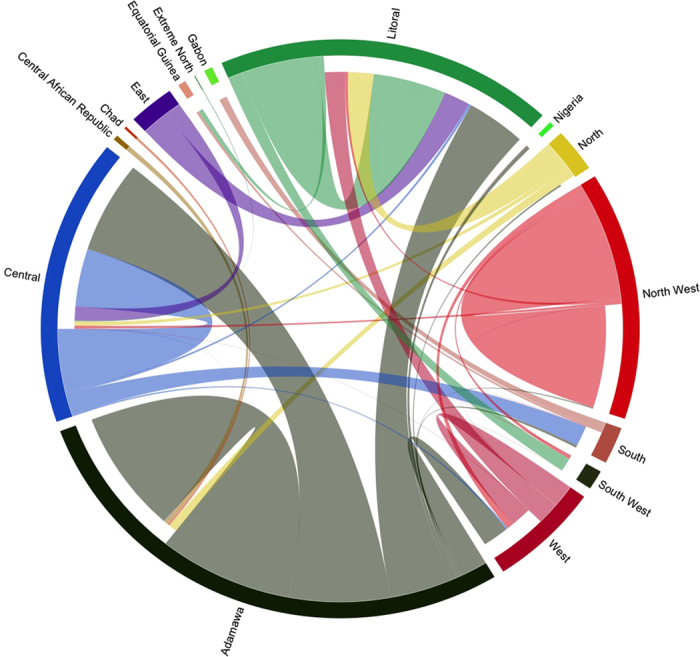
Cattle flow in the livestock market system in Cameroon. The origin and destination of traded cattle are represented in this circular map. Each sector of the circle represents a Region of Cameroon or a neighboring country involved by the cattle flow in the region. Outgoing animal flow starts from the base of each sector while received movements do not reach the base of the relevant sector. For instance, the North-West Region, in red, trades most of its animals within the same Region and only very small proportion of them are moved to the Littoral, Central, West and South-West Regions of Cameroon or are introduced from the Adamawa Region.

Over the annual period, between September 2013 and August 2014, 252, 831 individual cattle were traded and moved through the 127 markets identified in the study. Figure 2 shows the organic and heterogeneous structure of the network with all the Regions of the country being interconnected and with various trading links with neighboring countries. The Adamawa and Central Regions of Cameroon have the most heterogeneous cattle movements structure, displaying connections with several other Regions and with neighboring countries. The Adamawa Region appears to be the main source of cattle of the country while receiving animals from neighbouring countries, such as Chad and Central African Republic. In contrast, the Littoral and Central Regions appear to be major receivers of cattle, getting animals from almost all the other areas of the country. Interestingly, the North-West Region appears to be more independent and isolated within the cattle trade network of Cameroon, particularly receiving few animals from others Regions.

**Figure 3:**
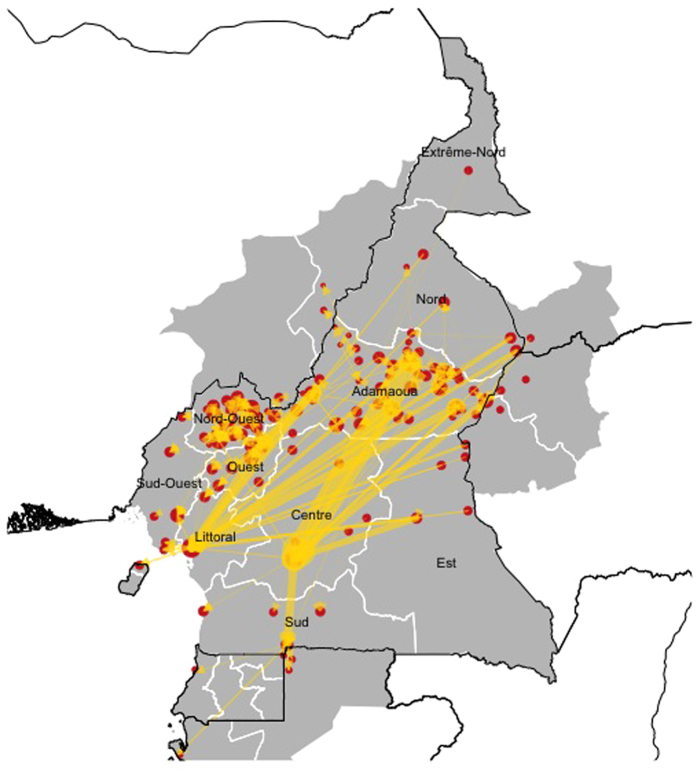
Cattle trading network in Cameroon and neighbouring areas. Node sizes in the map are weighted by the relative value of eigenvector centrality. Ties show the direction of the cattle movement as indicated by the arrows and the proportional volume of traded animals is indicated by the thickness of the arrow (Generated using R statistical software^40^ (version 3.2.3) using the *raster, rgdal* and *ggplot2* packages, and shp files obtained from the GADM database of Global Administrative Areas (source: www.gadm.org)^49^.

**Figure 4:**
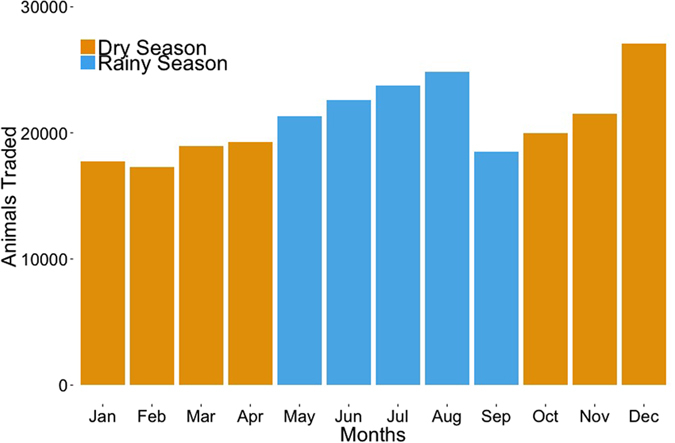
Volume of cattle traded through the network by month over a 12 month period. Blue bars refer to the rainy season (May to September) while yellow bars refer to the dry (October to April). Between September 2013 and August 2014, a total of 252,831 cattle were moved through the network.

Due to the constant weekly nature of the market chain, all nodes were consistently part of the network over the year, with little variation in network connectivity between seasons. The number of links ranged from 328 during the rainy season (May to September), to 333 in the dry season (October to April). For the whole 12-month period, the network included 335 links (Fig. 3). The main variation between seasons was related to the average number of animals traded per month (Fig. 4): on average, the number of traded cattle during the rainy season was 1.1 times greater than that traded during the dry season. Nevertheless, the greatest number of traded cattle was recorded in December (Fig. 4).

Over the entire study period, both network-level and node-level metrics showed little variation between seasons. As such, subsequent analyses were carried out over the annual aggregated network. The network displayed a low density (0.021) of connections, with only 2.1% of the possible links present. The reciprocity of the network was 0.151, indicating that just 15.1% of the connections among markets were reciprocal, or bidirectional. The degree assortativity was −0.06: such a negligible negative value indicates a minimal tendency for markets to preferentially connect with markets of different degree centrality. A minimum of eight steps were required for connecting the two most distant reachable nodes, as measured by the network diameter. The visualization of the actual path of the diameter showed that it extended from Chad, across the Adamawa Region of Cameroon ending in the North-West Region at the border with Nigeria (see Supplementary Section D).

### Network topology

The average path length and the clustering coefficients of the empirical cattle trade network in Cameroon were 3.07 and 0.25, respectively. In comparison, the average path length of 1000 simulated random networks was 4.74 (range = 4.39–5.38), whereas the 90*th* percentile of CC was below 0.05, five times lower than the empirical cattle trade network. These network characteristics are consistent with a small-world structure^41^,^42^. On average, markets were connected to 5.2 others (range 1–33), with 3.2 average incoming (range 0–29) and 2.6 outgoing (range 0–11) connections. The degree distribution for all markets involved in the cattle trade network was right-skewed with only a small proportion of markets being highly connected (20% of the markets held 58% of the connections, while 80% of the markets held the other 42% connections) (See Supplementary Section E).

### Key-actors analysis

**Figure 5:**
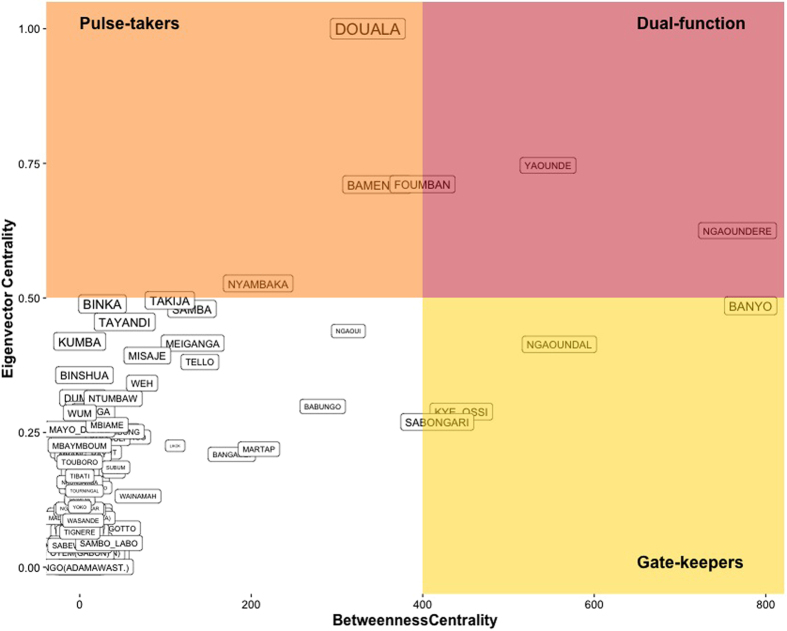
Key-actors analysis on the markets network. Correlation between eigenvector centrality and betweenness centrality is identifying markets with different roles within the trading network. “Gate-keepers”, in the bottom-right quadrant, are central entities in terms of their ability to bridge between the functional nodes of the network and wider community of nodes. In the top-left quadrant, “pulse-takers” are the nodes with the shortest paths to all other nodes having easy access to the other central markets as well as to the rest of the network. Nodes in the top-right quadrant have both abilities. Markets in the bottom left quadrant tend to have no particular role. The size of the labels is relative to the value of their residuals obtained through linear regression of betweenness over eigenvector centrality, indicating the extent of their deviation from a linear relationship.

All the studied centrality measures, in- and out-degree, eigenvector centrality and betweenness centrality were strongly positively correlated (Spearman correlation *r* > 0.7) (See Supplementary Section F). The smallest correlation coefficient resulted between eigenvector centrality and betweenness centrality (*r* = 0.70) and was applied to detect markets not having a linear relation between these metrics. Ten markets were identified as critical in the network: four were defined as the nucleus for structural functionality of the network (“pulse-takers”), four as being fundamental in connecting parts of the network that would otherwise be isolated from the network (“gate-keepers”), and two markets as having both these attributes (Fig. 5).

### Cohesive analysis

The cattle trade network of Cameroon was organised around five cores (respectively 23, 18, 16, 16 and 13 nodes) and involved a periphery of 41 markets that were connected to the different cores (see Supplementary Section G). All 127 markets involved in the network were included in the giant weak component. In contrast, 98 strongly connected components were identified. Of these strong components, 88 contained only one node, nine ranged between two and seven, while the largest included 11 markets, representing therefore 10% of all markets. The GSCC extended across the Adamawa and North Regions of Cameroon (see Supplementary Section G).

When looking at the community structure of the network, 15 communities were identified within the network. Each community included between two and 19 markets. The two largest communities included 19 markets each, therefore accounting for nearly 30% of all markets. The largest community involved markets located in four Regions of Cameroon (Adamawa, Central, North-West and West) and a market in the Taraba State of Nigeria (see Supplementary Section F). The second largest community involved 18 markets within the North-West Region and one market in the South West Region of Cameroon, thereby confirming the cohesion and relative isolation of the North-West Region of Cameroon (Fig. 2 and Supplementary Section G).

### Network vulnerability and resilience

**Figure 6:**
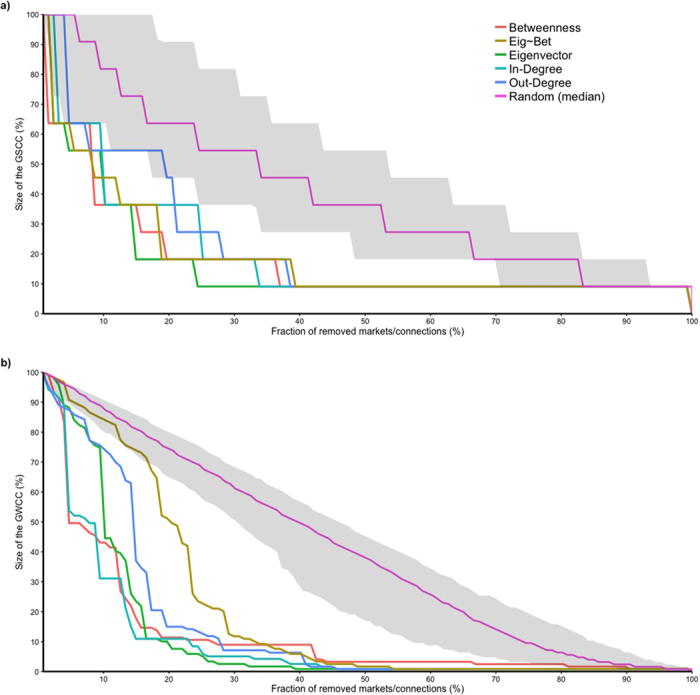
Effectiveness of targeted removal of nodes and connections over the GSCC (**a**) and the GWCC (**b**) in the cattle trade network. The y axis shows the size of the largest strong (**a**) and weak (**b**) component expressed as a percentage and the x axis shows the percentage of nodes, or connections, removal from the network. The effect of node removal, driven by the different centrality measures on the fragmentation of the components, is shown by the different colors: betweenness centrality in red, eigenvector in dark green, in-degree in light blue, out-degree in purple and the residuals from the regression of betweenness over eigenvector centrality in light green. Effect of link removal depending on their edge betweenness scores is showed by the light orange line. Random removal of nodes over 1000 simulations is showed with the median value (pink line) and its 95% range (gray shaded area).

**Figure 7:**
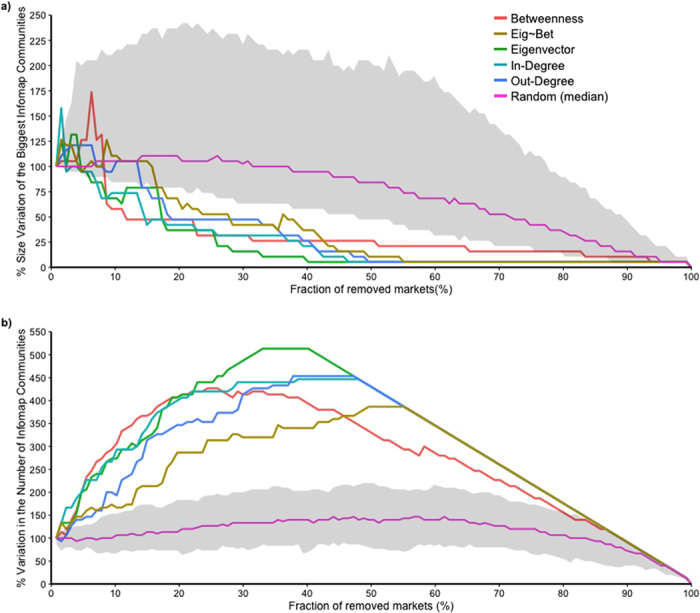
Effectiveness of targeted removal of nodes over the size of the biggest communities (**a**) and over the number of communities (**b**) in the cattle trade network. The y axis shows the percentage variation in the size of the biggest communities (**a**) and in the number of communities (**b**) of nodes within the network and the x axis shows the percentage of node removal. The effect of node discharge, driven by the different centrality measures on the number of communities present in the network, is shown by different colors: betweenness centrality in red, eigenvector in light orange, in-degree in light blue, out-degree in purple and the residuals from the regression of betweenness over eigenvector centrality in light green. Random removal of nodes over 1000 simulations is shown by the median value (pink line) and its 95% range (gray shaded area).

Results of the percolation analysis on the cohesion of the network structure are shown in Figs 6 and 7. Removing markets based on their centrality measures resulted in notably faster changes in network structure than randomly targeting markets: with a faster reduction in the size of the GSCC, GWCC and the biggest community, as well as a faster increase in the number of communities involved in the network.

Removing markets in the order of their values of betweenness, eigenvector and according to the key-actor analysis were the most effective strategy to fragment the GSCC: targeting 20% of the most central nodes reduced the size of the GSCC by approximately 80% (Fig. 6a). While betweenness and the results of the key actor analysis seemed to be slightly more effective during the early stages of disruption, eigenvector was overall leading to a more effective fragmentation at later stages. Eigenvector, betweenness together with in-degree were also the most effective in disrupting the GWCC reducing its size by about 90% through the removal of around 20% of the nodes (Fig. 6b). The size of the biggest community was reduced by about 70% through the removal of around 25% of nodes driven by eigenvector, betweenness and in-degree (Fig. 7a). Node removal driven by these same metrics triggered the highest fragmentation of the network leading to a four-fold increase in the number of communities targeting around the 25% of the markets (Fig. 7b).

## Discussion

The presence of formal livestock traceability systems that electronically record movements of animals between premises currently enables the description of livestock trade networks in numerous high income countries. This information provides the opportunity to investigate the features associated with livestock movements and disease spread, improving animal health monitoring and surveillance systems^1^,^2^,^11^,^13^,^43^,^44^. In lower income countries, however, the structure and dynamics of livestock trade networks are still poorly understood despite being critical to rural household livelihoods^12^,^14^,^15^,^45^,^46^. In SSA, where some of the world’s most rapid urbanization is taking place^7^, the restricted availability of data from regions with extensive and small-holder livestock systems limits the understanding of livestock movements. In this study, we described for the first time the network of cattle trade between markets in Cameroon. Notably, we have evaluated key markets in the trade of cattle in Cameroon and showed that markets in both neighbouring and non-neighbouring countries are epidemiologically connected via long-distance and cross-border cattle trade routes. These findings are critical for better designing targeted monitoring, surveillance, control and communication strategies against animal diseases and improving the sustainability of the livestock trade system in Cameroon.

Over the study period, there was a clear seasonality in the flow of cattle in the network, with more cattle traded during the rainy season (that is between April and August) than during the dry season. This pattern is likely due to the higher abundance of pasture during the rainy season, allowing animals to be fatter and therefore more marketable. However, the high volume of cattle traded in Cameroonian markets in December, despite being in the middle of the dry season, suggests that traditional behaviors and religious celebrations at the end of the year could be an important factor influencing the demand for animals on the market. Nevertheless, there was little variation in the structural characteristics of the cattle trade network as well as in its properties across seasons, showing that, despite the seasonality in traded numbers, the network of cattle moving between markets in Cameroon is very stable. The consistent structure of the network over the year increases the robustness of targeted interventions to temporal variations.

This study has characterized the Cameroonian cattle trade network as a compact network with multiple subgroups of markets which are interconnected through the presence of a few long distance connections typical of a small-world network. In addition, we found that any two markets in the cattle trade network of Cameroon are reachable through an average of 3.1 steps, whereas only eight steps (as informed by the network diameter) are necessary to get from one side of the network to the other. While small-world networks are known to ease the spread of infectious diseases, thereby favouring rapid dissemination of pathogens through the network^11^,^19^,^21^,^47^, such a small network diameter is of concern. Examining the actual pathway of the diameter, which extends from Chad to Nigeria through the Adamawa and North-West Regions, it is clear that there is a potential direct connection between non-neighbouring countries, allowing diseases to spread rapidly across the region. These findings highlight that neither large geographical distances nor national boundaries represent a barrier to the dissemination of infectious diseases in this part of Africa. In this context, national strategies are likely to have limited effectiveness if developed alone and in isolation and that regional coordination for designing and implementing prevention and mitigation strategies against infectious diseases is essential to improve animal health in SSA.

Nevertheless, the cattle trade network in Cameroon is heterogeneous and organised around regional clusters of trading behaviours. As a major cattle production area in Cameroon, it is not surprising that markets from the Adamawa and Central Regions are highly connected with the rest of the country and with the neighbouring countries, forming not only the largest community of contacts of the cattle trade network but also includes 10 out of the 11 markets involved in the GSCC. In contrast, the North-West Region of Cameroon was found highly isolated to the rest of the network, with cattle preferably moving between markets within this region and forming a large community of contacts. Nonetheless, a few incoming and outgoing trade connections with other areas of the country or with foreign countries allowed markets of the North-West Region to remain connected with the rest of the network. These findings suggest that the Adamawa and Central Regions may be at higher risk for disease incursions and rapid dissemination over long distances, while the North-West Region could be more protected from the introduction of external threats, however, being more prone to a rapid local dissemination of pathogens. This Region, therefore, may also provide a more manageable setting for piloting the efficacy of surveillance or control measures compared with other Regions of the country.

The targeted removal of highly connected nodes is considered an effective method to identify nodes where the implementation of intervention measures for surveillance and control strategies, such as implementing trade restrictions, vaccination or animal testing, would be most efficient^26^. Here, we found that targeting the top 20% of the most connected markets (as defined by their eigenvector, betweenness centrality and the outcome of the key-actors analysis) would significantly reduce the network cohesiveness, regardless of the pathogen being directly transmitted between markets via animal movements or indirectly through personnel, vehicles or infrastructures. Although these findings provide opportunities for targeted disease risk mitigation interventions based on these connectivity measures, such a strategy may be difficult to implement on the ground. While market closure has been suggested as a highly effective strategy in interrupting the dissemination of an infection over the network^45^, such a mitigation approach could potentially lead to unintended effects. Particularly, the potential severe damages in profits, for both private and public stakeholders, due to the closure of important trading markets may create novel pattern of cattle movements (either legal or illegal) within the country. The resulting rewired cattle trade network could lead to an increase in the risk of disease dissemination as well as to reduce the control of the veterinary services over cattle trade patterns. Alternative disease risk mitigation interventions could be the implementation of increased bio-security measures, introduction of preventive emergency vaccinations for high risk animals in combination (or not) with quarantine measures, and movements bans for cattle originating from high-risk areas. These have the advantage to conserve the pattern of cattle trade network while requiring limited technical and financial resources as well as ensuring the collaboration of all actors of the cattle industry in Cameroon.

Targeting key markets as part of surveillance strategies has the potential to increase the detection sensitivity of diseases, particularly compared with randomly implemented interventions. Improving early detection of diseases would decrease the risk of importing, spreading and exporting infectious diseases at a local and regional level. Simultaneously, could increase cost-efficiency of interventions enabling better allocation of resources for animal health interventions. It is worth noting that targeting key markets of the cattle trade network for communication and training campaigns has the potential to raise the awareness of stakeholders present in these markets regarding infectious diseases risks and livestock management practices. Raising awareness could ideally facilitate the involvement of market stakeholders (i.e. farmers, herders, traders) in a “passive” surveillance system for early detect of newly introduced diseases as well as of endemic pathogens, thereby also facilitating data collection. Such a framework could be especially important for key priority livestock and zoonotic diseases in the region such as foot and mouth disease (FMD), lumpy skin disease (LSD), contagious bovine pleuro-pneumonia, bovine tuberculosis, brucellosis and Rift Valley fever.

In Cameroon, as in most of SSA, traders (i.e. herdsmen with cattle to sell or buy) and dealers (known locally as “buy’m sell’m”) whose primary activity is dealing cattle between markets, are the key actors in the cattle marketing system. Commonly, traders and dealers buy and sell cattle locally, only visiting few markets within a restricted area because most cattle movements still occur on foot and in order to limit expenses and optimise their profits. In a minority of cases, however, some dealers are buying larger numbers of cattle at sub-regional and regional markets to sell at national markets (located near major urban areas) for slaughter. In both situations, however, individual animals will not always be immediately sold and those unsold animals may move back to the herd of origin (i.e. by traders) or be sold at the same market on another sale day or to another market (i.e dealers). In this study, the cattle trade network between markets in Cameroon was informed by the official market records of cattle transactions (purchases and sales). As such, it is reasonable to believe that the number of animals moving from and to markets may be underestimated, which may affect some of our results. However, given the costs of holding cattle and moving between markets there are limited economic incentives to retain unsold animals for dealers and therefore this underestimation should be limited. In addition, because the structure of the trading system is very stable in Cameroon, we believe that our study captures the structure (the nodes and the links) of the network well. Furthermore, other studies on the effects of incomplete information on the properties of empirical networks have shown that eigenvector centrality and in-degree were robust to incomplete information, even when 50% of the data were missing^48^.

This study aimed to describe and analyse the livestock trade network in Cameroon. However, it was not possible for security reasons to physically visit and sample the North and Extreme North Regions of the country. As such, we are aware that this study missed an established direct pathway of cattle trade, known to flow from high livestock production regions in Central Africa towards countries with a high demand of animal and animal products in West Africa passing through the Extreme-North Region of Cameroon. In this study, we focused on the movement of cattle among markets, regardless of the type of animals moved. However, there are additional pathways that may determine pathogen transmission between markets, including movements of other animals, people and vehicles. By focusing on the trading system we only targeted the formal cattle movements in the country missing the role of the informal seasonal cattle transhumance and underestimating the scale of both the across-country and cross-border cattle movements, and the more organic and complex nature of livestock interactions at a local, national and regional level.

In settings where there is still restricted information on livestock movements, formal quantitative approaches based on empirical observations and analyses are increasingly needed to understand the interactions between livestock production and trade systems, pathogen transmission and the wider environment. As the design of risk-based surveillance systems and of control and communication strategies increasingly requires prior epidemiological knowledge, this study aimed at providing the baseline for more effective evidence-based decisions and interventions for diseases where live animal trading constitutes a major risk for disease introduction and dissemination. The major outputs of this paper show that the cattle trade network in Cameroon (1) is very stable throughout the year, (2) has a very clear small-world structure with long-distance connections and clustered areas which are isolated to others, and (3) presents the opportunity for targeted surveillance, control and communication interventions. Despite the limitations of the present project and the need for further studies on cattle population dynamics in the region, the identification of key livestock markets and long-distance interactions as well as the characterization of the various roles of the Regions of Cameroon within the cattle trade system could offer the opportunity for “intelligent tracing”, applying empirical knowledge to infectious disease communication, surveillance and prevention.

## Additional Information

**How to cite this article:** Motta, P. *et al*. Implications of the cattle trade network in Cameroon for regional disease prevention and control. *Sci. Rep.* **7**, 43932; doi: 10.1038/srep43932 (2017).

**Publisher's note:** Springer Nature remains neutral with regard to jurisdictional claims in published maps and institutional affiliations.

## Supplementary Material

Supplementary Material

## Figures and Tables

**Table 1:** Network-level and node-level metrics.

Measures	Definition
**Network-level metrics**	
*Density*	Number of edges in the observed network relative to the total number of possible edges in a completely connected network
*Reciprocity*	Proportion of edges showing mutual connections (Measure of the tendency of vertex pairs to form mutual connections between each other)
*Degree Assortativity*	Quantifies the tendency of individual nodes to connect with other nodes which are similar to themselves in terms of degree centrality
*Average path length*	Average number of edges that must the traversed to connect any two nodes in the network, without accounting for the temporal dimension of the connections
*Diameter*	The shortest distance/path length between the two most distant connected nodes in the network ( = the longest of all the calculated path lengths).
*Clustering coefficient (CC)*	Proportion of pairs of neighbors of a given node which are connected, measures the tendency of the network to cluster
*Giant strongly connected components (GSCC)*	The largest subset of nodes that are mutually reachable through directed paths
*Giant strongly connected components (GWCC)*	The largest subset of nodes that are mutually reachable through undirected paths, therefore not accounting for the directionality of connections
**Node-level metrics**	
*In-degree*	Computed accounting for both the number of connections that each node receives in a defined period and the weights of these connections, hence a measure of the potential sources or origins of infection in that range of time
*Out-degree*	Computed accounting for both the number of connections that each node sends in a defined period and the weights of these connections, and therefore the number of potential destinations of infection in that range of time.
*Betweenness centrality*	Frequency with which a node is located on the shortest path length between any pairs of nodes, accounting that the connection between nodes might be stronger along paths with more intermediate nodes that are strongly connected than paths with fewer weakly-connected links. In other words, it is a measure of the tendency of connecting nodes which would be otherwise disconnected
*Eigenvector centrality*	Indirect measure of centrality determined by the centrality scores of the nodes to which the node of interest is connected
**Key actors**	
*Gate-keepers*	Central nodes in terms of their ability to bridge between the functional basis of the network and the wider community of nodes. They are characterized by high betweenness centrality and low eigenvector centrality values
*Pulse-takers*	Represent the functional basis of the network, are nodes with the shortest paths therefore easy access to other central nodes as well as to the rest of the network. They are characterized by high eigenvector centrality and low betweenness centrality values
*Dual functionality*	Nodes that fulfil the same bridging role as gate-keepers simultaneously having easy access to all areas of the network. They are characterized by both high eigenvector centrality and betweenness centrality values
