# Supplementary material for: Implications of the cattle trade network in Cameroon for regional disease prevention and control
Source: Sci Rep. 2017 Mar 7;7:43932. doi: 10.1038/srep43932 (PMC5339720; doi:10.1038/srep43932)
Supplement: Supplementary Material [file srep43932-s1.pdf]

Implications of the cattle trade network in Cameroon for regional disease prevention and control

Paolo Motta[1,2*], Thibaud Porphyre[1], Ian Handel[2], Saidou M. Hamman[3], Victor Ngwa[4], Vincent Tanya[5], Kenton Morgan[6], Rob Christley[7,8], and Barend M. deC. Bronsvoort[1,2]

[1]*The Roslin Institute, University of Edinburgh, Easter Bush Campus, EH25 9RG, United Kingdom*

[2]*Royal (Dick) School of Veterinary Science, University of Edinburgh, Easter Bush Campus, EH25 9RG, United Kingdom*

[3]*Institute of Agricultural Research for Development, Regional Centre of Wakwa, Ngaoundere, Cameroon*

[4]*School of Veterinary Medicine and Sciences, University of Ngaoundere, Ngaoundere, B.P. 454, Cameroon*

[5]*Cameroon Academy of Sciences, Yaoundé, B.P. 1457, Cameroon*

[6]*Institute of Ageing and Chronic Diseases, University of Liverpool, Leahurst Campus, CH64 7TE, United Kingdom*

[7]*Institute of Infection and Global Health, University of Liverpool, Leahurst Campus, CH64 7TE, United Kingdom*

[8]*NIHR Health Protection Research Unit in Emerging and Zoonotic Infections, University of Liverpool, Liverpool, L69 3BX, United Kingdom*

[*]*paolo.motta@roslin.ed.ac.uk*

A: Data Collection

The proportions of markets listed within the official registers and present within the study Regions of the Adamawa, West and North-West (n=52), and of markets identified through the preliminary analysis of the official reports located within these three Regions (n=7) were overall consistent across the three Regions of the study area (Figure S1). This data collection approach enabled us to identify a total of 59 cattle markets within the Adamawa, West and North-West Regions where active data collection was carried out (Figure S1) and 68 markets outside these Regions. Furthermore, 68 markets were identified outside these three study Regions: 46 were listed by MINEPIA in Cameroon (including the 3 national markets located in Yaounde and Douala, the two major urban centres of Cameroon, and in Kye-ossi, a border town in the South Region neighbouring with Gabon and Equatorial Guinea); 10 were identified by local veterinarians to be within Cameroon and an additional 13 markets in the 5 neighbouring countries.

B: Data Description

Figure S2A shows the proportion of the data that was provided by official reports and the proportion obtained by the interviews with the veterinary officials. More than 60% of these reports were complete, including data

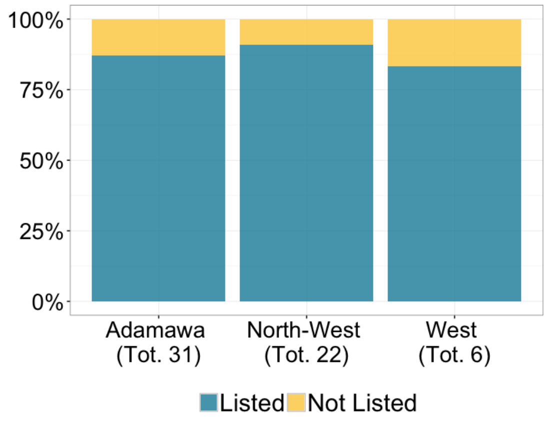

Figure S1: **Proportions of markets identified through the official list and though the investigation within the three main study Regions.** Blue colour refers to the proportion of the markets that were officially listed while the yellow colour refers to the markets that were not on this lists.

on the number of traded animals, their origins and destinations for the whole 12 month period (Figure S2B). With regards to the origins and destinations of the traded cattle, among this 60% of reports with complete data the 92% were also consistent with the information gathered through the interviews with the veterinary officials (Figure S2C). When the documents were complete information was prioritized from this source while when this data source was not complete the missing information was obtained from the interviews. In the restricted proportion of cases for which both official documentation and survey data were available and not matching (8% of the cases in which complete documentation was available) the official source was prioritized compared to the results of the interview.

C: Community detection method

Community detection was carried out to identify communities of markets which tend to be closely connected while less connected with nodes of other communities. As the framework for the detection of the structure of communities in networks is still relatively under development and as the detection methods should be used in combination with the empirical knowledge about the network under investigation, different community detection methods were tested. Edge betweenness method (Girvan and Newman, 2001), eigenvectors of matrices method (Newman, 2006), Infomap method (Rosvall and Bergstrom, 2008) and spectral clustering method based on stochastic block models (Rohe et al., 2011) were used for the community detection analysis. According to the local knowledge and to our expertise of Cameroon, among all the tested methods the Rosvall-Bergstrom identified the community structure that more closely reproduces the geographical localization of the markets and their connectedness on the territory, particularly considering natural physical barriers and regional/cultural communities and the observed connectedness between markets in the field. Results of this method are shown in Section G of this Supplementary Material (Figure S7).

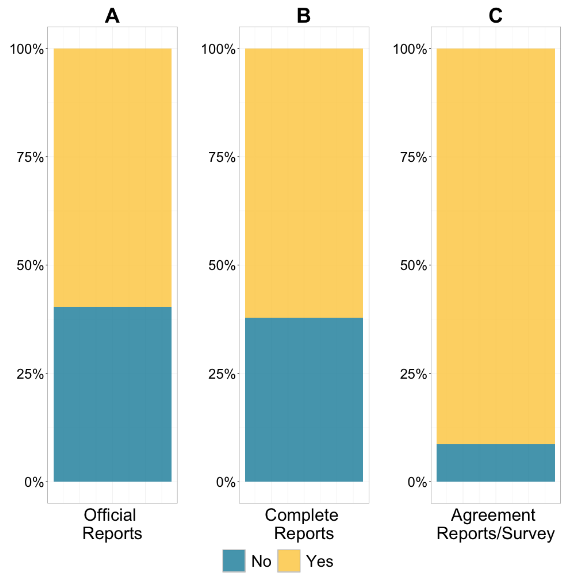

Figure S2: **Proportions of data on destinations and origins that were provided by the different data sources.** Figure A shows the proportion of information on sources and destination of cattle that was obtained from the official reports compared to the proportion from the interviews with the veterinary officials (Yes = from official reports; No = not from official reports). About 60% of these information could be obtained from the official reports and of this, 66% of these were complete records while 34% of the reports were incomplete (Figure B). Among the complete documents 92% of the reported information were consistent with the one obtained from the interviews (Figure C).

D: Network Diameter

Figure S3 shows the diameter off the network stretching across the center of Cameroon.

E: Nodes degree and degree distribution

As reported in the main manuscript node degree showed a right-skewed distribution of the trading connections (Figure S4).

The distribution of the trading connections identifies a small pool of markets which are highly connected (20% of the markets held 58% of the connections, while 80% of the markets held the other 42% connections)

F: Correlation between centrality measures

In Table S1 are shown the Spearman correlation coefficients among the centrality measures for the annual directed weighted network. We find that all the node centrality measures are highly correlated (Spearman r >0.7), with the smaller correlation between eigenvector centrality and betweenness centrality.

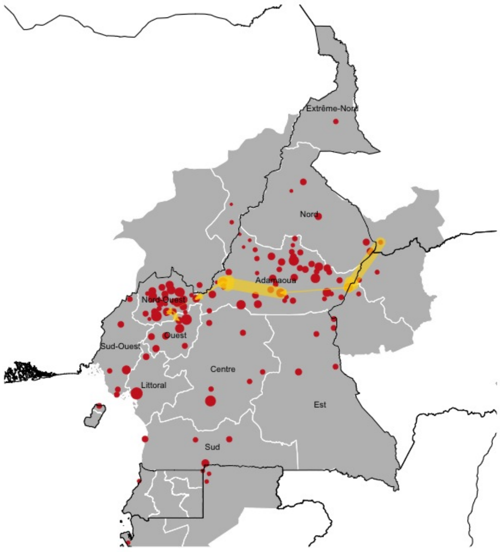

Figure S3: **Diameter of the trade network.** The pathways between the two most distant reachable markets in the network is extending from Chad through Central African Republic, crossing central Cameroon, passing close to the Nigerian border and ending in the North-West Region (Generated using R statistical software (version 3.2.3) using the *raster, rgdal* and *ggplot2* packages, and shp files obtained from the GADM database of Global Administrative Areas (www.gadm.org)).

	Betweenness	Degree	Eigenvector
Betweenness	1	0.8078	0.7087
Degree	0.8078	1	0.8879
Eigenvector	0.7087	0.8879	1

Table S1: Correlation coefficients between nodes centrality measures.

G: Cohesive subgroup analysis

Methods

Different cohesive analysis approaches were used in this study. Network component analysis was applied to assess the network overall connectivity accounting for the direction of the market connections, while a core-periphery analysis was performed to determine which markets formed a densely connected core and which are part of a sparsely connected periphery. Community detection analysis, instead, was carried out to identify groups of more densely connected markets (Section C of this Supplementary Material).

Results

While the identified GWCC included all the markets of the trade network the GSCC contained only about 10% of the markets in the network (Figure S6). The GSCC is stretching across two Regions of Cameroon, the Adamawa and the North Regions.

Core-periphery analysis characterised a network periphery composed of 41 nodes and five cores (respectively 23, 18, 16, 16 and 13 nodes) (Figure S6). As the definition by Borgatti and Everett (2000) describes a

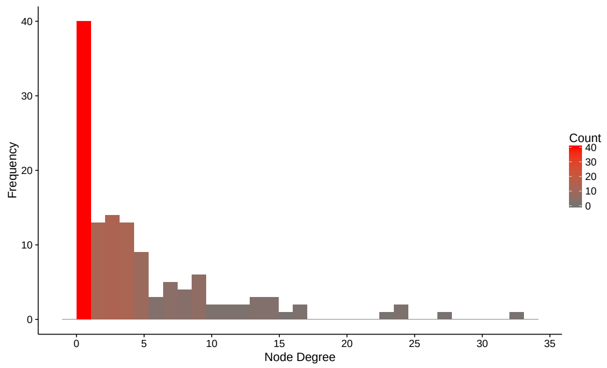

Figure S4: **Degree distribution of the livestock trade network.** Degree distribution for the 127 livestock markets included in the network between September 2013 and August 2014.

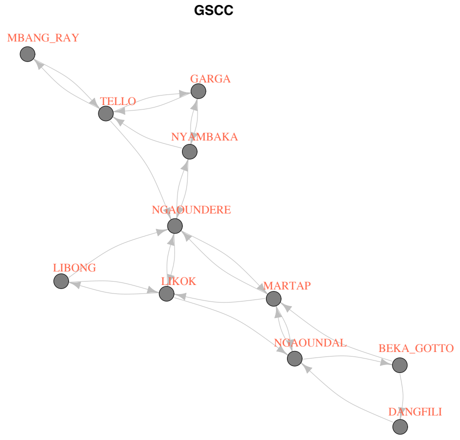

Figure S5: **Giant strong connected component of the livestock trade network.** The GSCC included 11 livestock markets mainly located in the Adamawa Region, and one in the North region of Cameroon.

core as a center with many cores and a peripheral community which is connected to the different cores, this analysis defined the composition of these two subgroups of markets within the network.

Community detection identified 15 market communities within the network: 3 main communities of almost consistent dimension (2 communities of both 19 nodes and a community of 17 nodes) and other communities of gradually decreasing size (Figure S7).

References

Adams, L., Gray, G. and Murray, G. Animal biosecurity in the Mekong: future directions for research and development. In Adams L.B., G. G. and G, M. (eds.) Proceedings of an international workshop held in Siem Reap, Cambodia, 10–13 August 2010, 45–59 (2012).

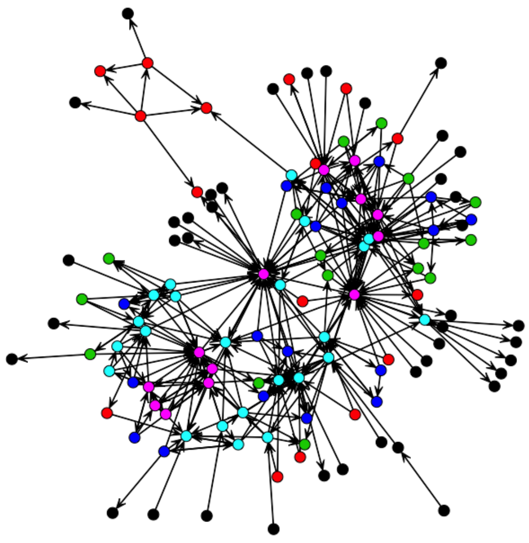

Figure S6: **Core-Periphery structure of the livestock trade network.** The periphery of the network included 41 markets (in black) while 5 cores composed of respectively 13, 24, 26, 19 and 22 markets were identified.

Clauset, A., Rohilla, C. and Newman,S.M.E.J. Power-Law Distributions in Empirical Data. SIAM Rev. 51, 661-703 (2009).

Conway, D. Analyzing Terrorist Networks - Theories and Techniques (2010). URL http://riskecon.com/wp-content/uploads/2012/02/Conway-SocioTerrorism.pdf.

Dawson, P. M. et al. Epidemic predictions in an imperfect world: modelling disease spread with partial data. Proceedings. Biological sciences / The Royal Society 282, 20150205 (2015).

Gile, K. J. and College, N. Improved Inference for Respondent-Driven Sampling Data with Application to HIV Prevalence Estimation (2010).

Gillespie, C. S. Fitting heavy tailed distributions: the poweRlaw package (2014).

Girvan m. and Newman M. E. J. Community structure in social and biological networks. Proceedings of the National Academy of Sciences (12) 7821-7826 (2002).

Lancichinetti, A. and Fortunato, S. Community detection algorithms A comparative analysis. Physical Review E 80, 056117 (2009).

Moradi, F., Olovsson, T. and Tsigas, P. An Evaluation of Community Detection Algorithms on Large-Scale Email Traffic. 283-294 (Springer Berlin Heidelberg, 2012).

Newman, M. E. J. Finding community structure in networks using the eigenvectors of matrices. Physical Review E 74, 036104 (2006)

Rosvall, M. and Bergstrom, C. T. Maps of random walks on complex networks reveal community structure. Proceedings of the National Academy of Sciences 105, 1118–1123 (2008).

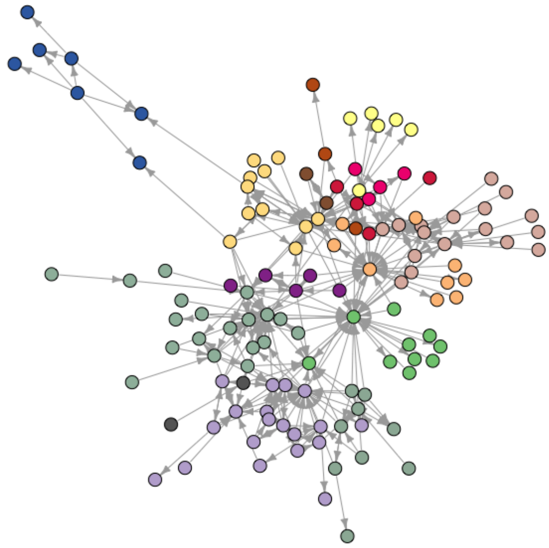

Figure S7: **Communities of the livestock trade network.** The 15 different nodes communities are highlighted with different colors.

Salganik, M. J., Heckathorn, D. D. and Heckathornt, D. D. Sampling and Estimation in Hidden Populations Using Respondent-Driven Sampling. Source: Sociological Methodology 3421547 (2004).
